# Amyloid Precursor Protein Abnormalities Destabilize Membrane Ferroportin: A Novel Mechanism Underlying Early Brain Pathologies and Memory Impairment in Alzheimer’s Disease

**DOI:** 10.3390/ijms27093892

**Published:** 2026-04-27

**Authors:** Yifan Xiao, Wenli Huang, Lingyan Chen, Rufeng Huang, Yuhui Guo, Wei Liu, Xiaochuan Wang, Jianzhi Wang, Jian Bao, Xiji Shu

**Affiliations:** 1 Hubei Key Laboratory of Cognitive and Affective Disorders, School of Medicine, Jianghan University, Wuhan 430056, China; yfxiao@jhun.edu.cn (Y.X.); 17862890905@163.com (W.H.); chenlingyan011120@163.com (L.C.); 19872056252@163.com (R.H.); 19921392475@163.com (Y.G.); liuwei@jhun.edu.cn (W.L.); wxch@mails.tjmu.edu.cn (X.W.); wangjz@mail.hust.edu.cn (J.W.); 2 Department of Pathology and Pathophysiology, School of Medicine, Jianghan University, Wuhan 430056, China; 3 Institutes of Biomedical Sciences, School of Medicine, Jianghan University, Wuhan 430056, China

**Keywords:** Alzheimer’s disease (AD), *ferroptosis*, *ferroportin* (FPN), *amyloid precursor protein* (APP), memory impairment

## Abstract

Alzheimer’s disease (AD) research has primarily focused on amyloid beta (Aβ) and *tau* protein; however, drug development targeting these two proteins has been disappointing. Therefore, there is an urgent need to explore the novel pathogenic mechanisms underlying AD. Recently, we found that expression of the *K670N/M671L*-mutated amyloid precursor protein (APP) in 293T cells significantly reduced membrane ferroportin (FPN) levels. Furthermore, 2-month-old *APP/PS1* mice exhibited a marked decrease in membrane FPN levels, while total FPN expression and Aβ levels remained unchanged. Further studies revealed that features of ferroptosis were present in the brains of 2-month-old *APP/PS1* mice, and that treatment with ferroptosis inhibitors or iron chelation significantly alleviated early pathological changes and cognitive impairment in these animals. In addition, supplementation with an APP–FPN binding peptide during the early phase ameliorated AD-related pathologies, including Aβ deposition, neuroinflammation, oxidative stress, and synapse-associated protein deficits, in *APP/PS1* mice. Collectively, our findings suggest that APP mutations may contribute to early brain pathological changes and subsequent memory impairment in AD by downregulating membrane trafficking of FPN and inducing ferroptosis, thereby providing new molecular targets for drug development.

## 1. Introduction

Alzheimer’s disease (AD), the most prevalent neurodegenerative disorder worldwide, is characterized by progressive cognitive decline and synaptic dysfunction, imposing a substantial burden on global healthcare systems and families [1]. AD is characterized by two pathological hallmarks: the accumulation of intracellular neurofibrillary tangles (NFTs) formed by abnormally hyperphosphorylated tau proteins, and extracellular senior plaques composed of aggregated amyloid-β (Aβ) [2]. After abnormal hyperphosphorylation of the tau protein, it will lose its original function, and then aggregate to form tangles, damaging the structure and function of nerve cells [3]. When the balance between the production and clearance of the Aβ protein is broken, it will deposit outside the cells and form plaques, triggering a series of pathological reactions. Over the past few decades, the Aβ cascade hypothesis posits that the deposition of the Aβ protein is the pivotal initiating event in Alzheimer’s disease, triggering a cascade of pathological reactions that ultimately result in neuronal death and cognitive decline [4]. Based on this hypothesis, numerous studies have focused on developing drugs and therapeutic strategies aimed at clearing Aβ plaques [5]. Conversely, the tau hyperphosphorylation hypothesis underscores the significant role of abnormal tau protein hyperphosphorylation in disease progression, suggesting that inhibiting tau hyperphosphorylation and tangle formation is crucial for treating Alzheimer’s disease [6]. Consequently, most therapeutic strategies have targeted the removal of Aβ plaques and the suppression of tau tangles, with the hope of ameliorating patients’ conditions through these approaches [7,8,9]. Despite extensive efforts, nearly all clinical trials based on these classical theories have yielded disappointing outcomes, failing to reverse or halt the relentless progression of AD. This has spurred researchers to persistently delve into novel research avenues and therapeutic strategies, with the aim of discovering more efficacious treatment modalities and instilling hope in patients [10,11].

Emerging evidence has demonstrated that the maintenance of iron metabolic homeostasis is the core premise to ensure the normal physiological function of neurons [12]. It not only participates in the synthesis and secretion of neurotransmitters and mitochondrial energy metabolism, but plays an irreplaceable role in the construction and maintenance of synaptic plasticity [13,14]. The abnormal accumulation of free iron and the disorder of redox balance caused by iron overload in the brain have been clearly confirmed to be closely related to the onset [15] and progression of a variety of neurodegenerative diseases [16,17], such as Alzheimer’s disease (AD), Parkinson’s disease (PD), brain iron deposition neurodegenerative disease, and so on [18]. Ferroportin (FPN) is the only known non-heme iron exporter protein in mammalian cells at present [19]. This protein can mediate the transmembrane afflux of divalent iron in cells [20], cooperate with ceruloplasmin, membrane iron transport auxiliary protein [21], and other molecules to complete the iron transport and metabolic cycle, and play a key pivotal role in regulating iron efflux and maintaining iron homeostasis in the central nervous system [22]. Ferroptosis is a new type of regulatory cell death driven by iron dependent lipid peroxidation chain reaction, which is different from traditional apoptosis and necrosis [23,24]. Many studies have confirmed that ferroptosis is involved in the pathophysiological processes of a variety of neurodegenerative diseases, yet its precise role and underlying molecular regulatory mechanism in AD pathogenesis remain largely elusive [24,25].

Ferroportin (FPN), the only known iron exporter, is a multi-pass transmembrane protein characterized by twelve transmembrane domains and glycosylation sites. It plays an irreplaceable core role in maintaining intracellular iron homeostasis, regulating iron efflux rate and preventing abnormal iron accumulation [26]. Its transcription, translation, post-translational, and membrane localized Amyloid precursor protein (APP) is a type I transmembrane glycoprotein full chain expression, are strictly regulated at multiple levels, precisely and reversibly, so as to dynamically adapt to cellular iron demand and to maintain central and peripheral iron balance [27]. At the transcriptional level, FPN is modulated by factors such as iron deficiency, hypoxia, and inflammatory cytokines [28]. At the translational level, it is regulated by the iron responsive element/iron regulatory protein (IRE/IRP) system located in the 5′ untranslated region [29,30]. At the post-translational level, FPN undergoes hepcidin-mediated ubiquitination [31]. Recent studies have highlighted that the stability of FPN at the plasma membrane is critical for maintaining cellular iron homeostasis [32,33,34].

Amyloid precursor protein (APP) is a type I transmembrane glycoprotein. APP is processed through two distinct secretase pathways. Soluble amyloid precursor protein (sAPPα) is the product of the non-amyloidogenic pathway mediated by α-secretases (ADAM family) [35] Extensive research has indicated that *s*APP*α* exerts significant neuroprotective effects, promoting neuronal survival, enhancing synaptic plasticity, and acting as a neurotrophic factor [36]. By contrast, *s*APP*β* is the soluble ectodomain generated by the cleavage of APP via β-secretase (BACE1), representing the amyloidogenic pathway. Although *s*APP*β* itself does not form plaques, its generation signifies that APP is being channeled toward Aβ production. The cleavage by BACE1 is the rate-limiting step in Aβ generation; consequently, the level of *s*APP*β* is widely recognized as a biomarker for BACE1 activity and amyloid burden. The accumulation of sAPPβ indicates an increased production of downstream Aβ peptides (Aβ*40/*Aβ*42*), which leads to amyloid plaque deposition and neurotoxicity [37].

The extracellular region contains a ferroportin targeting peptide (FTP) [21], corresponding to amino acids 327–348 of APP695, which is the core sequence responsible for the binding of APP to the iron output protein (FPN). The α-secretase-cleaved fragment of APP (sAPPα) significantly enhances its binding to FPN, thereby increasing the stability of FPN at the plasma membrane [38]. By contrast, the β-cleaved fragment (sAPPβ) reduces this binding and subsequently decreases membrane FPN stability. These findings suggest that conformational changes in APP may affect the interaction between its FTP domain and FPN [39,40], thereby modulating the membrane stability of FPN and participating in the regulation of neuronal iron transport [41].

## 2. Results

### 2.1. The Mutation of APP (K670N/M671L) Caused the Decrease of FPN on Cell Membrane

To perform the predictive analysis, we download the high-resolution FPN and APP protein models from the UniProt protein database (https://www.uniprot.org/, accessed on 12 March 2022), remove the water, hydrogen, and other ligands in PyMOL 3.1 software, and import the protein model files into the HDOCK platform for molecular docking to obtain the top 100 results of binding energy. We then select the docking result with the highest binding energy, check the confidence score and resolution, and then perform visualization processing in PyMOL 3.1 software. Finally, we query and analyze the specific site binding information.

**Figure 1:**
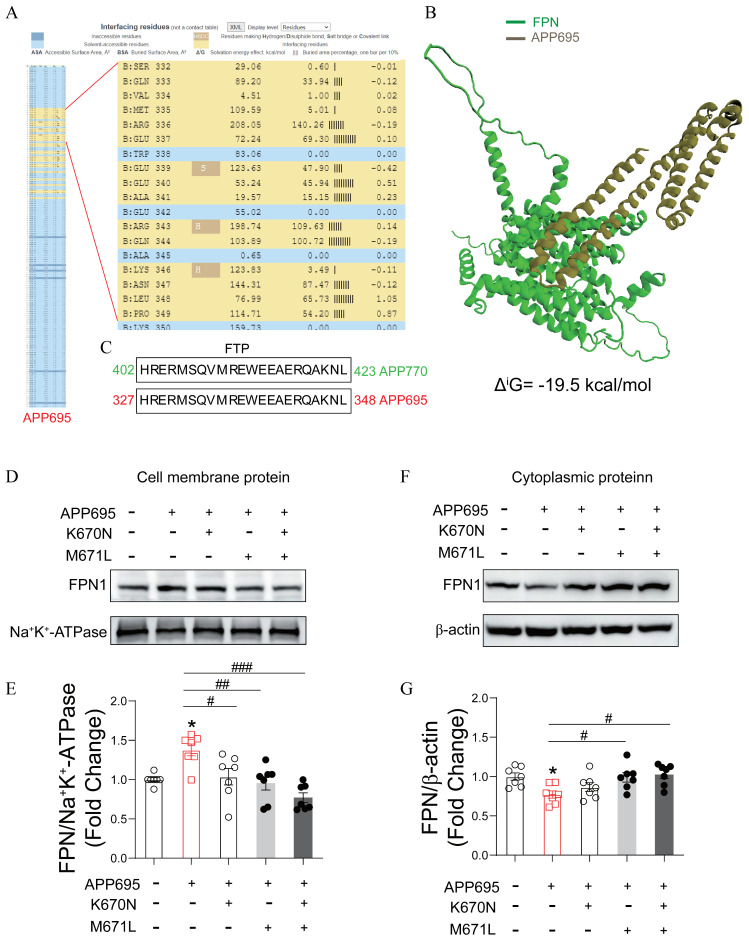
The *K670N/M671L* mutation in APP disrupted FPN interaction and reduced membrane trafficking. (**A**) Predicted APP region that interacts with FPN. (**B**) Detailed analysis of interface residues involved in the interaction between APP695 and FPN. (**C**) APP695 regions reported to bind FPN in multiple studies. (**D**) Western blot analysis of membrane FPN levels in SY5Y cell lines overexpressing wild-type APP695, APP K670N, or/and M671L mutants. (**E**) Quantification of membrane FPN levels normalized to Na^+^/K^+^-ATPase. All data were normalized to the vehicle group and are presented as mean ± SEM (*n* = 7 per group). Statistical significance was determined using one-way ANOVA. * *p* < 0.05 vehicle vs. APP695; # *p* < 0.05, ## *p* < 0.01, ### *p* < 0.001, APP695 vs. mutants. (**F**) Western blot analysis of cytosolic FPN levels in 293Tcell lines expressing wild-type APP695, APP K670N, or/and M671L mutants. (**G**) Quantification of cytosolic FPN levels normalized to β-actin. All data were normalized to the vehicle group and are presented as mean ± SEM (*n* = 7 per group). Statistical significance was determined using one-way ANOVA. * *p* < 0.05 vehicle vs. APP695; # *p* < 0.05, ## *p* < 0.01, ### *p* < 0.001, APP695 vs. mutants.

Our predictive analysis using software indicated a strong binding affinity between APP and FPN (Figure 1A,B). According to the relevant literature, APP can interact with FPN, and the interacting domain has been termed FTP (Figure 1C). The lysine-to-asparagine substitution at position 670 (*K670N*) and methionine-to-leucine substitution at position 671 (*M671L*) of APP, collectively known as the Swedish mutation, represent one of the most well-characterized pathogenic variants associated with familial AD. However, it remains unclear whether this dual mutation alters the binding capacity of APP for FPN. To further validate the impact of these mutations on the APP–FPN interaction, we introduced APP mutations at the cellular level and observed changes in FPN localization on the cell membrane. As shown in Figure 1D,E, overexpression of wild-type APP in 293T cells increased membrane-bound FPN compared to the vehicle group. Mutation of either *M670N* or *K671L* in APP resulted in a slight decrease in membrane-associated FPN, whereas simultaneous mutation of both residues led to a marked reduction compared to the wild-type APP group. Correspondingly, cytoplasmic FPN levels were decreased in the wild-type APP group compared to the vehicle group, whereas increased levels were observed following *M670N* and *K671L* mutations relative to the wild-type APP group, suggesting that mutations at positions 670 and 671 impair the membrane trafficking of FPN (Figure 1F,G). These findings provide strong evidence that the 670/671 mutations in APP suppress FPN localization to the plasma membrane.

**Figure 2:**
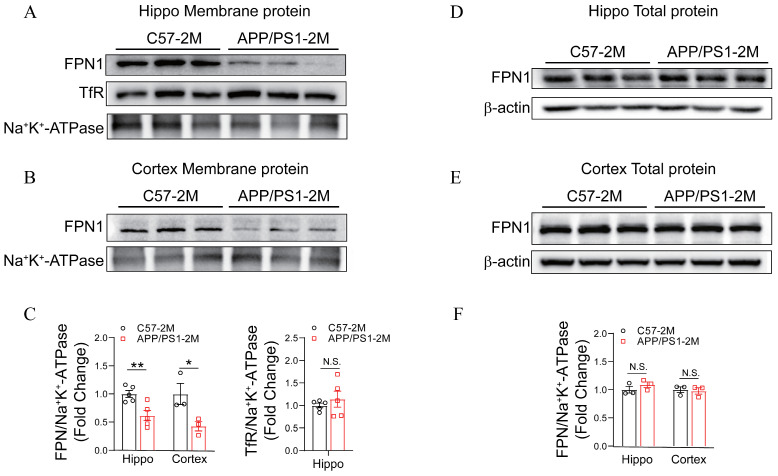
The *K670N/M671L* mutation in APP reduced FPN trafficking to cell membrane in 2-month-old *APP/PS1* mice. Membrane FPN and TfR levels in (**A**) the hippocampus and (**B**) the cortex of 2-month-old *C57BL/6J* and *APP/PS1* mice were assessed by western blotting. (**C**) Quantification of membrane FPN levels in the mouse brain was normalized to Na^+^/K^+^-ATPase. Data are expressed as mean ± SEM (*n* = 3 mice per group). * *p* < 0.05, ** *p* < 0.01 (unpaired two-tailed Student’s *t*-test). Total FPN levels in (**D**) the hippocampus and (**E**) the cortex of 2-month-old *C57BL/6J* and *APP/PS1* mice were assessed by western blotting. (**F**) Quantification of total FPN levels in the mouse brain was normalized to β-actin. Data are expressed as mean ± SEM (*n* = 3 mice per group, unpaired two-tailed Student’s *t*-test). N.S. (unpaired two-tailed Student’s *t*-test).

To further investigate the effect of APP mutations on FPN expression on cellular membrane, we utilized 2-month-old *APP/PS1* transgenic mice, which carried human APP (Swedish) and PS1 (ΔE9) mutations, leading to Aβ plaque deposition, neuroinflammation, and cognitive deficits. The membrane and cytoplasmic protein extraction kit (Beyotime Biotech Inc., Shanghai, China) was used to isolate membrane and cytoplasmic proteins separately. Our observations revealed that, compared to 2-month-old *C57BL/6J* mice, 2-month-old *APP/PS1* mice exhibited a significant reduction in membrane-associated FPN in the hippocampus and cortex, while the level of transferrin receptor (TfR) remained unchanged (Figure 2A–C). Interestingly, total FPN protein levels in the hippocampus and cortex were unchanged in 2-month-old *APP/PS1* mice, compared to that from 2-month-old *C57BL/6J* mice (Figure 2D–F). These findings indicate that APP mutations primarily impair the membrane trafficking of FPN without altering its overall expression level.

### 2.2. APP Mutation Results in Iron Deposition in APP/PS1 Mice

**Figure 3:**
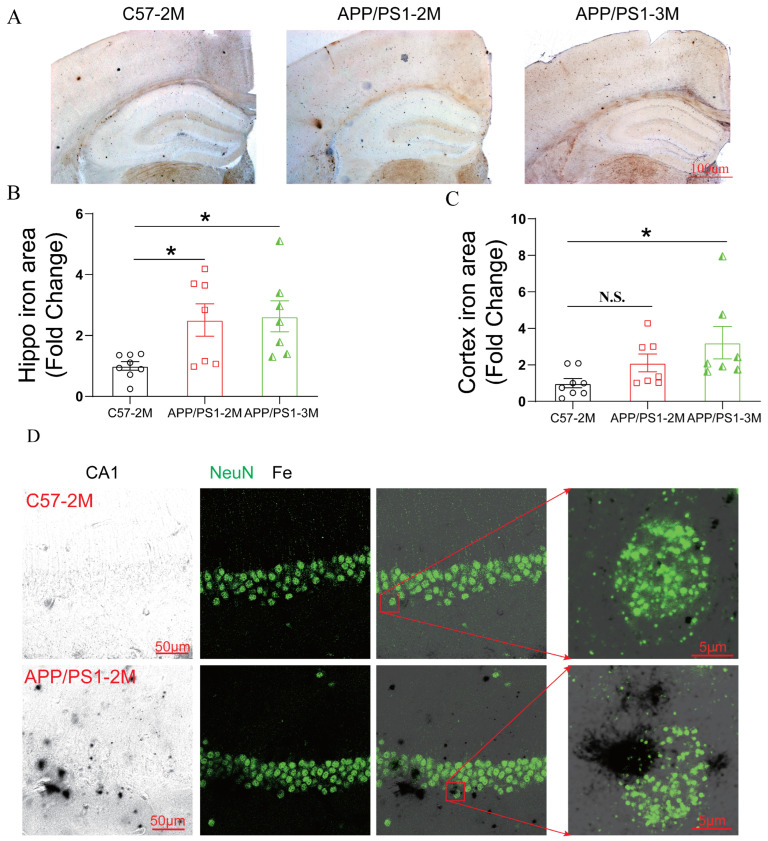
Iron was overloaded in the hippocampus of 2-month-old *APP/PS1* mice. (**A**) Representative images of iron staining in the brain of *C57BL/6J* control mice at 2 months of age (*C57BL/6J*-2M) and *APP/PS1* transgenic mice at 2 months (*APP/PS1*-2M) and 3 months (*APP/PS1*-3M) of age. Scale bars = 100 μm. Quantification of iron area from (**B**) the hippocampus and (**C**) the cortex and presented as fold change relative to *C57BL/6J*-2M mice. Data are expressed as mean ± SEM (*n* = 7–8 mice per group). * *p* < 0.05 (one-way ANOVA). (**D**) Representative images of iron staining and neuron (NeuN) in CA1 from *C57BL/6J*-2M and *APP/PS1*-2M. Scale bars = 50 μm or 5 μm (enlarged images). N.S. (unpaired two-tailed Student’s *t*-test).

Given that FPN is the sole iron exporter and the expression of TfR remained unchanged, we hypothesized that the reduction in membrane-bound FPN in the brains of *APP/PS1* mice likely leads to iron accumulation during the early phase. To test this, we measured iron levels and found that, compared with 2-month-old *C57BL/6J* mice, 2-month-old *APP/PS1* mice exhibited significant iron accumulation in the hippocampus as early as 2 months of age, whereas iron levels in the cortex remained unchanged (Figure 3A,B). Meanwhile, iron accumulation became more pronounced in 3-month-old *APP/PS1* mice (Figure 3C). Furthermore, immunofluorescence staining confirmed prominent iron accumulation within neurons in the brains of 2-month-old *APP/PS1* mice (Figure 3D). Notably, by 6 months of age, iron levels showed a decreasing trend compared with those in 3-month-old *APP/PS1* mice (Figure S1A,B).

These findings suggest that 2 months of age represents a critical time window for the onset of iron elevation in the brain, with levels peaking at 3 months and subsequently plateauing or declining, possibly attributable to the involvement of other regulatory factors in vivo.

### 2.3. APP Mutation Triggers Ferroptosis in the Early Stage of APP/PS1 Mice

**Figure 4:**
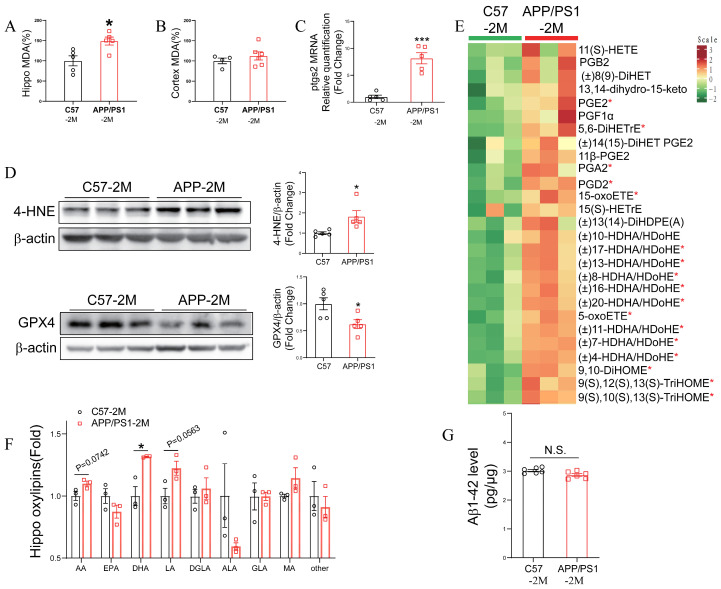
Lipid peroxidation increased in the hippocampus of 2-month-old *APP/PS1* mice. Quantification of malondialdehyde (MDA) from (**A**) the hippocampus and (**B**) the cortex in *C57BL/6J* control mice and *APP/PS1* transgenic mice at 2 months of age. Data are expressed as mean ± SEM (*n* = 4–6 mice per group). * *p* < 0.05 (unpaired two-tailed Student’s *t*-test). (**C**) Relative mRNA expression of *ptgs2* (fold change) in the hippocampus. Data are expressed as mean ± SEM (*n* = 4–5 mice per group). *** *p* < 0.001 (unpaired two-tailed Student’s *t*-test). (**D**) 4-HNE and GPX4 in the hippocampus of 2-month-old *C57BL/6J* and *APP/PS1* mice were assessed by western blotting. Quantification of 4-HNE and GPX4 levels was normalized to β-actin. Data are expressed as mean ± SEM (*n* = 5 mice per group). * *p* < 0.05 (unpaired two-tailed Student’s *t*-test). (**E**) Heatmap depicting the relative levels of unsaturated fatty acid metabolites from the hippocampus of 2-month-old *C57BL/6J* and *APP/PS1* mice was displayed. * *p* < 0.05 (unpaired two-tailed Student’s *t*-test). (**F**) Quantification of oxyphosphates (fold change) of various fatty acids in the hippocampus, including arachidonic acid (AA), eicosapentaenoic acid (EPA), docosahexaenoic acid (DHA), linoleic acid (LA), dihomo-γ-linolenic acid (DGLA), α-linolenic acid (ALA), γ-linolenic acid (GLA), and myristic acid (MA), among others. Data are expressed as mean ± SEM (*n* = 3 mice per group). * *p* < 0.05 (unpaired two-tailed Student’s *t*-test). (**G**) Quantification of Aβ_1–42_ levels in the hippocampus (pg/g tissue) from the hippocampus of 2-month-old *C57BL/6J* and *APP/PS1* mice. Data are expressed as mean ± SEM (*n* = 3 mice per group). N.S. (unpaired two-tailed Student’s *t*-test).

Given that increased iron levels may trigger ferroptosis in vivo through downstream unsaturated lipid peroxidation, we next examined ferroptosis-related markers in the brains of 2-month-old *APP/PS1* mice. Compared with 2-month-old *C57BL/6J* mice, 2-month-old *APP/PS1* mice exhibited a significant increase in the lipid peroxidation product MDA in the hippocampus (Figure 4A), whereas no obvious change was observed in the cortex (Figure 4B). This finding aligns with the region-specific iron accumulation described above, which was more pronounced in the hippocampus. Further, we measured the mRNA level of the ferroptosis marker prostaglandin-endoperoxide synthase 2 (*ptgs2*) in the hippocampus and found that it was already markedly elevated in the hippocampus of 2-month-old *APP/PS1* mice, compared to 2-month-old *C57BL/6J* mice (Figure 4C).

More specifically, compared with 2-month-old *APP/PS1* mice, a marked increase in 4-hydroxynonenal (4-HNE), a major end-product of lipid peroxidation, was observed, alongside a significant reduction in the antioxidant enzyme glutathione peroxidase 4 (GPX4) (Figure 4D). Furthermore, a metabolomic analysis revealed a substantial increase in multiple unsaturated fatty acid metabolites in the hippocampus of 2-month-old *APP/PS1* mice, compared to that from 2-month-old *C57BL/6J* mice (Figure 4E), primarily derived from arachidonic acid (AA), linoleic acid (LA), and docosahexaenoic acid (DHA) (Figure 4F and Figure S2A–D). However, no significant increase in Aβ levels was observed at this stage (Figure 4G).

**Figure 5:**
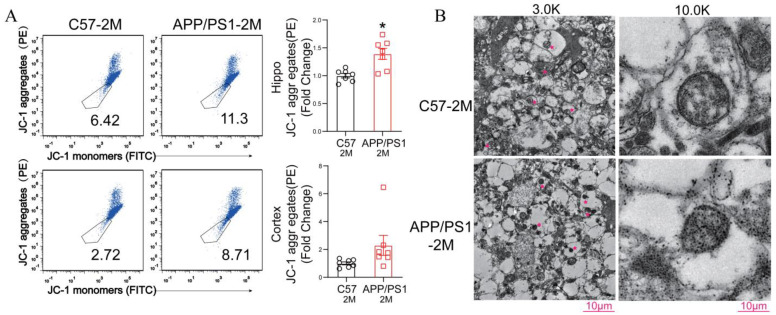
Mitochondria were damaged in the hippocampus of 2-month-old *APP/PS1* mice. (**A**) Representative flow cytometry analysis of JC-1 monomers and aggregates staining in the hippocampus of 2-month-old *C57BL/6J* control mice and 2-month-old *APP/PS1* transgenic mice. Data are expressed as mean ± SEM (*n* = 7 mice per group). * *p* < 0.05 (unpaired two-tailed Student’s *t*-test). (**B**) Representative electron microscopy images of the hippocampal sections from 2-month-old *C57BL/6J* and *APP/PS1* mice. Scale bars = 10 μm.

Moreover, ferroptosis is typically characterized by distinct mitochondrial morphological changes, including membrane damage, volume shrinkage, increased membrane density, and reduction or loss of cristae. Therefore, we next examined mitochondrial alterations and found that the hippocampus of 2-month-old *APP/PS1* mice exhibited an increase in JC-1 aggregates, suggesting enhanced mitochondrial membrane damage, compared to that from 2-month-old *C57BL/6J* mice (Figure 5A). Electron microscopy further revealed mitochondrial shrinkage and increased electron density in the hippocampus of 2-month-old *APP/PS1* mice (Figure 5B), providing additional evidence of significant mitochondrial injury at this early stage.

Collectively, these findings demonstrated that ferroptosis occurs prior to Aβ deposition in the early stages of Alzheimer’s disease, suggesting that inhibition of ferroptosis may represent a promising therapeutic strategy.

### 2.4. Inhibition of Ferroptosis and Iron Chelation in the Early Stage Alleviated Later-Stage AD-like Pathologies in APP/PS1 Mice

**Figure 6:**
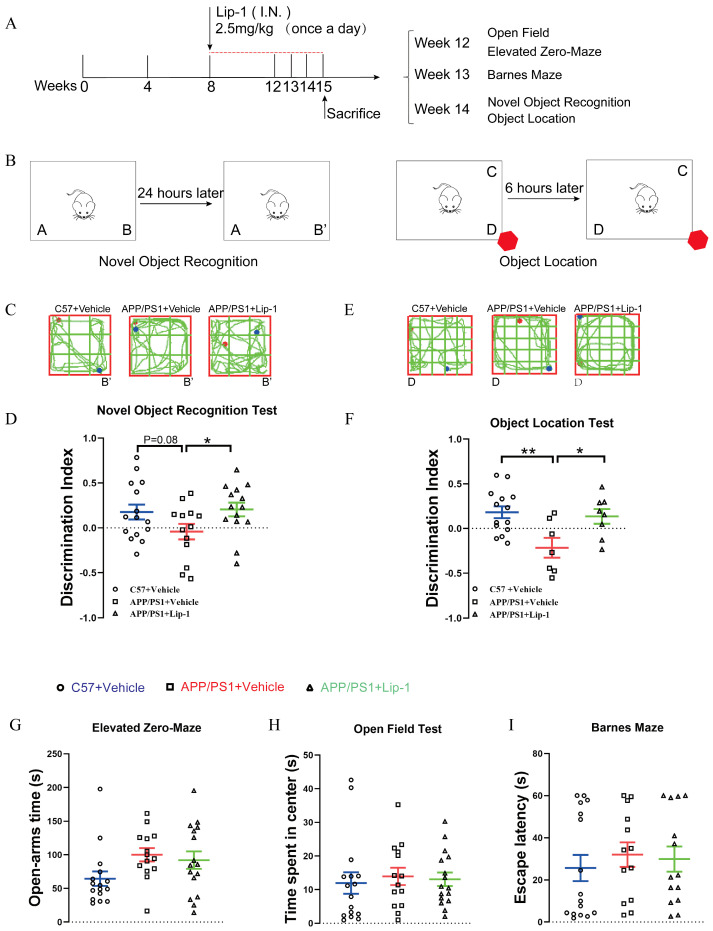
Lip-1 treatment at an early stage improved cognitive and behavioral deficits in *APP/PS1* mice. (**A**) Schematic of the treatment protocol. Lip-1 (2.5 mg/kg, once daily) or vehicle was administered intranasally to 2-month-old *C57BL/6J* control and *APP/PS1* transgenic mice. Behavioral tests were conducted one month after the start of treatment. (**B**) Schematic diagram of the novel object recognition test and quantification of the discrimination index in *C57BL/6J* + Vehicle, *APP/PS1* + Vehicle, and *APP/PS1* + Lip-1 groups. (**C**) Schematic diagram of the object location test and quantification of the discrimination index in the three groups. Quantification of the exploratory activity in the elevated zero maze (**D**), time spent in the center during the open field test (**E**), and escape latency during the Barnes maze test (**F**) in the three groups. Data are expressed as mean ± SEM (*n* = 7–14 mice per group). * *p* < 0.05, ** *p* < 0.01 (one-way ANOVA). (**G**) in the three groups. Data are expressed as mean ± SEM (*n* = 13–16 mice per group). * *p* < 0.05, (one-way ANOVA). (**H**) in the three groups. Data are expressed as mean ± SEM (*n* = 13–16 mice per group). * *p* < 0.05, (one-way ANOVA). (**I**) in the three groups. Data are expressed as mean ± SEM (*n* = 13–16 mice per group). * *p* < 0.05, (one-way ANOVA).

To further investigate the role of ferroptosis in the early pathology of *APP/PS1* mice, we administered a ferroptosis inhibitor (Lip1, 2.5mg/kg) at 2 months of age and assessed its effects on AD-related behavioral outcomes (Figure 6A). The results showed that, compared with untreated *APP/PS1* mice, ferroptosis inhibition significantly improved performance in the novel object recognition (Figure 6B) and object location recognition tests (Figure 6C) in *APP/PS1* mice treated with Lip1. However, no significant improvements were observed in spatial learning tasks, including the elevated zero maze (Figure 6D), open field tests (Figure 6E), and the Barnes maze (Figure 6F). These findings suggest that ferroptosis in early AD primarily contributes to the pathological changes underlying recognition and object-location memory.

**Figure 7:**
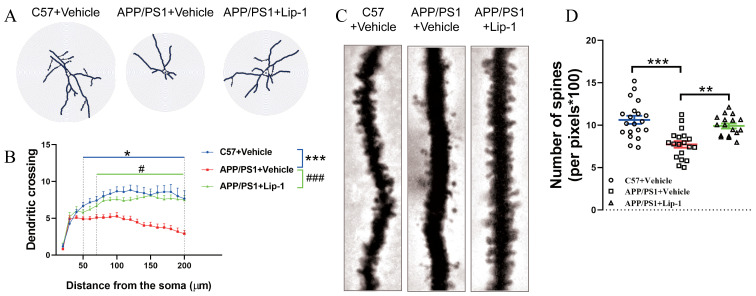
Lip-1 treatment at an early stage improved synaptic ultrastructure in *APP/PS1* mice. (**A**) Representative images of dendritic morphology in the hippocampus of *C57BL/6J* + Vehicle, *APP/PS1* + Vehicle, and *APP/PS1* + Lip-1 groups. (**B**) Quantification of dendritic cross-sectional area (μm^2^) as a function of distance from the soma (μm). Data are presented as mean ± SEM (*n* = 19 fields per group). * *p* < 0.05, *** *p* < 0.001 (*C57BL/6J* + vehicle vs. *APP/PS1* + vehicle), # *p* < 0.05, ### *p* < 0.001 (*APP/PS1* + vehicle vs. *APP/PS1* + Lip1), two-way ANOVA, *n* = 19 fields per group. (**C**) Representative images of dendritic spines in each group. (**D**) Quantification of spine density (number of spines per 100 μm) in *C57BL/6J* + Vehicle, *APP/PS1* + Vehicle, and *APP/PS1* + Lip-1 groups. Data are presented as mean ± SEM (*n* = 17–20 fields per group). * *p* < 0.05, ** *p* < 0.01, (one-way ANOVA).

Further examination revealed that, compared to untreated *APP/PS1* mice, inhibition of ferroptosis significantly improved synaptic ultrastructure in the mouse brain, as evidenced by increased postsynaptic density length (Figure 7A,B) and higher dendritic spine density (Figure 7C,D) in *APP/PS1* mice treated with Lip1. These findings strongly suggest that early intervention with ferroptosis inhibition effectively alleviates neuronal damage.

Consistent with these findings, administration of an iron chelator (M30, 0.5 mg/kg) (Figure S3A) to 2-month-old *APP/PS1* mice significantly improved their performance in the object location recognition test compared to untreated *APP/PS1* mice (Figure S3B).

The above results suggested that early inhibition of ferroptosis resulting from iron overload could effectively alleviate later-stage AD-like pathologies in *APP/PS1* mice.

### 2.5. Supplementation with the APP–FPN Binding Peptide Alleviates AD-Related Pathologies

**Figure 8:**
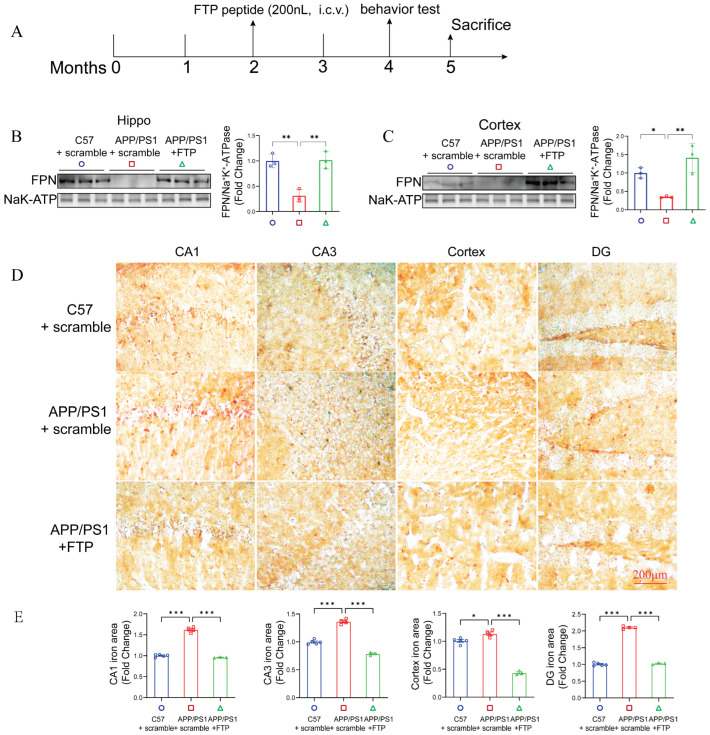
FTP supplementation reduced iron deposition in *APP/PS1* mice. (**A**) Schematic of the treatment protocol. Scramble or FTP (800 nM, 200 nL per side, bilateral) was administered via intracerebroventricular (i.c.v.) injection. Behavioral tests were conducted two months after the start of treatment. Membrane FPN levels in (**B**) the hippocampus and (**C**) the cortex of *C57BL/6J* + Scramble, *APP/PS1* + Scramble, and *APP/PS1* + FTP mice were assessed by western blotting. Quantification of membrane FPN levels in the mouse brain was normalized to Na^+^/K^+^-ATPase. Data are expressed as mean ± SEM (*n* = 3 mice per group). * *p* < 0.05, ** *p* < 0.01 (one-way ANOVA). (**D**) Representative images of iron staining in the brain of *C57BL/6J* + Scramble, *APP/PS1* + Scramble, and *APP/PS1* + FTP mice. Scale bars = 100 μm. (**E**) Quantification of iron area from the hippocampus and cortex. Data are expressed as mean ± SEM (*n* = 3–5 mice per group). * *p* < 0.05, *** *p* < 0.001 (one-way ANOVA).

Given that the 670/671 mutation in *APP/PS1* mice inhibits APP–FPN binding, leading to early iron accumulation, ferroptosis, and AD-like pathologies, we next sought to determine whether early restoration of FPN membrane levels could ameliorate AD-related deficits in vivo. To test this, we administered a peptide fragment of APP that binds FPN (FTP, 200 nL) through intracerebroventricular injection and evaluated behavioral changes after two month (Figure 8A).

A western blot analysis revealed that, compared to that from *APP/PS1* mice treated with scramble, administration with FTP peptide in *APP/PS1* mice significantly increased FPN levels in the hippocampal (Figure 8B) and cortex (Figure 8C) membrane, confirming the efficacy of the intervention. Next, we measured iron levels and found that, compared to the scramble group in *APP/PS1* mice, *APP/PS1* mice administered with FTP peptide exhibited significantly decreased iron accumulation in the hippocampus and cortex (Figure 8D,E).

**Figure 9:**
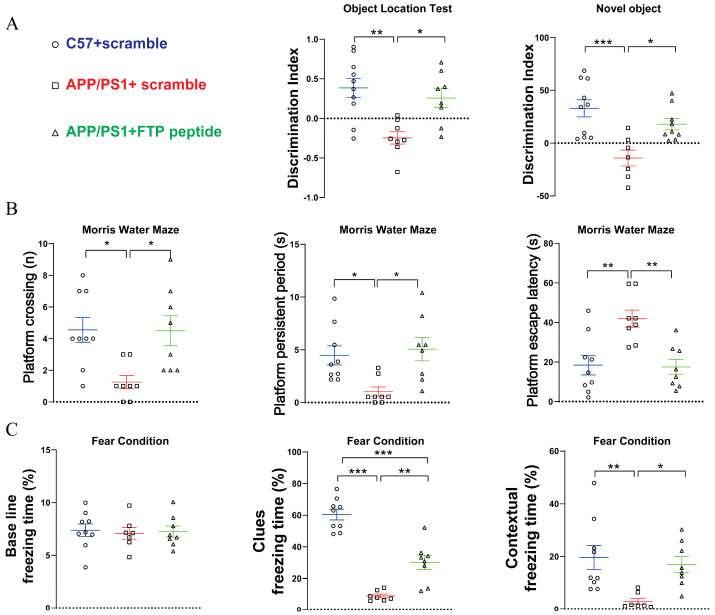
FTP supplementation alleviated AD-related learning and memory deficits in *APP/PS1* mice. (**A**) Quantification of the discrimination index in the object location test and novel object recognition test for *C57BL/6J* + Scramble, *APP/PS1* + Scramble, and *APP/PS1* + FTP groups. (**B**) Quantification of platform crossings (n), platform perseveration period (s), and escape latency (s) in the Morris water maze test for the three groups. (**C**) Quantification of baseline freezing time (%), cued freezing time (%), and contextual freezing time (%) in the fear conditioning test for the three groups. Data are expressed as mean ± SEM (*n* = 8–9 mice per group). * *p* < 0.05, ** *p* < 0.01, *** *p* < 0.001 (one-way ANOVA).

**Figure 10:**
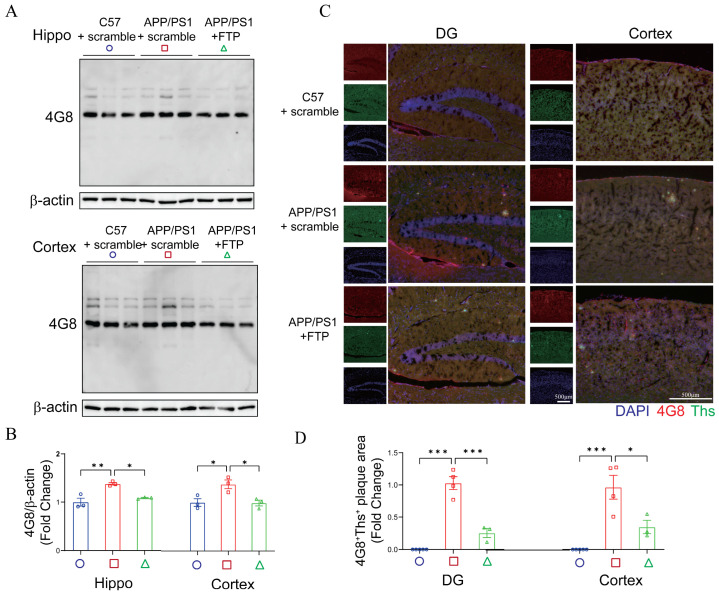
FTP supplementation reduced Aβ in *APP/PS1* mice. (**A**) 4G8 in the hippocampus and cortex from *C57BL/6J* + scramble, *APP/PS1* + scramble, and *APP/PS1* + FTP groups were assessed by western blotting. (**B**) Quantification of 4G8 in the mouse brain was normalized to β-actin. Data are expressed as mean ± SEM (*n* = 3 mice per group). * *p* < 0.05, ** *p* < 0.01 (one-way ANOVA). (**C**) Representative immunofluorescence staining of Aβ plaques (4G8, red) and Thioflavin S (Ths, green) in DG and cortex from *C57BL/6J* + scramble, *APP/PS1* + scramble, and *APP/PS1* + FTP groups. DAPI (blue) was used for nuclear counterstaining. Scale bars = 500 μm. (**D**) Quantification of 4G8^+^Ths^+^ Aβ plaques from DG and cortex. Data are shown as mean ± SEM (*n* = 3–4 mice per group). * *p* < 0.05, ** *p* < 0.01, *** *p* < 0.001 (one-way ANOVA).

**Figure 11:**
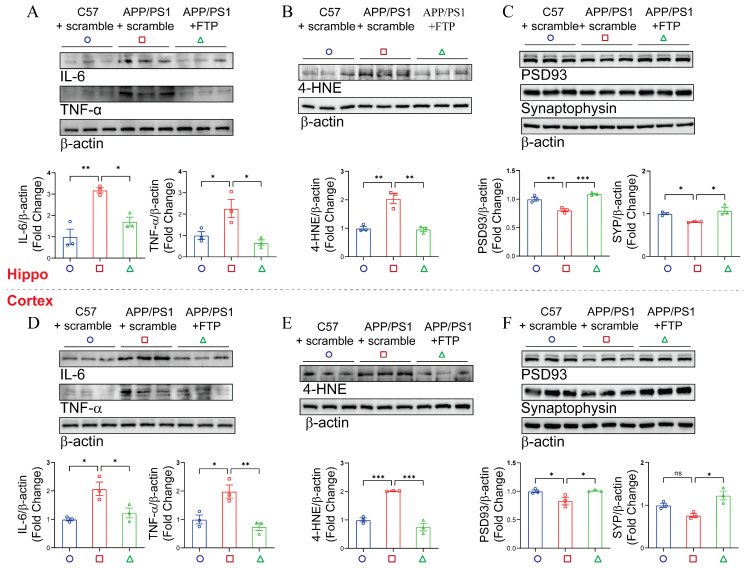
FTP supplementation attenuated neuroinflammation, oxidative stress, and synaptic protein dysfunction in *APP/PS1* mice. Representative western blot images of IL-6 and TNF-α (**A**), 4-HNE (**B**), PSD93 and synaptophysin (**C**) in the hippocampus of *C57BL/6J* + scramble, *APP/PS1* + scramble, and *APP/PS1* + FTP groups. Representative western blot images of IL-6 and TNF-α (**D**), 4-HNE (**E**), PSD93 and synaptophysin (**F**) in the cortex of *C57BL/6J* + scramble, *APP/PS1* + scramble, and *APP/PS1* + FTP groups. Quantification of IL-6, TNF-α, 4-HNE, PSD93, and synaptophysin expression were normalized to β-actin (fold change). Data are expressed as mean ± SEM (*n* = 3 mice per group). * *p* < 0.05, ** *p* < 0.01, *** *p* < 0.001 (one-way ANOVA). ns (unpaired two-tailed Student’s *t*-test).

Behavioral assessments revealed that FTP peptide treatment significantly improved learning and memory performance in *APP/PS1* mice, as evidenced by the object location recognition and novel object recognition (Figure 9A), as well as Morris water maze (Figure 9B) and fear conditioning tests (Figure 9C), compared to *APP/PS1* mice treated with scramble. Western blot (4G8, Figure 10A) and immunofluorescence (4G8 and Thioflavin S, Figure 10B) analyses further demonstrated that FTP treatment markedly reduced Aβ pathology, a hallmark of AD, in the hippocampus and cortex, compared to that from *APP/PS1* mice treated with scramble. Additionally, compared to *APP/PS1* mice treated with scramble, FTP treatment in *APP/PS1* mice significantly attenuated neuroinflammation (IL-6, TNF-a) (Figure 11A), oxidative stress (4-HNE) (Figure 11B), and synaptic proteins (PSD93, synaptophysin) (Figure 11C) in the hippocampus. A similar phenomenon was observed in the cortex (Figure 11D–F).

Collectively, these findings indicate that early intervention with the FPN-binding APP peptide—which corrects the reduction in membrane FPN caused by APP mutation—effectively alleviates AD-related pathologies in *APP/PS1* mice.

## 3. Discussion

The present study unravels a novel early pathogenic mechanism in AD, wherein mutant APP mediates the dysregulation of membrane FPN [39,41]. Specifically, we have demonstrated that *K670N/M671L*-mutated APP impairs FPN membrane localization (without altering total FPN expression) in both 293T cells and 2-month-old *APP/PS1* mice, by inducing conformational changes that weaken APP–FPN binding and cause aberrant cytosolic FPN retention. As the sole mammalian non-heme iron exporter, diminished membrane FPN disrupts iron efflux in the hippocampus and cortex [42,43], triggering early region-specific brain iron deposition (onset at 2 months, peak at 3 months), alongside ferroptosis hallmarks including elevated MDA levels, mitochondrial damage, and polyunsaturated fatty acid metabolic disorders [44,45]. This process precedes significant Aβ plaque deposition, confirming ferroptosis as an early initiating AD pathology [25,46]. Moreover, early targeted interventions, including ferroptosis inhibitor Liproxstatin-1, iron chelator M30, and APP–FPN binding FTP peptide, reverse membrane FPN downregulation, alleviate iron overload, restore synaptic structural damage, and ameliorate cognitive deficits in *APP/PS1* mice [47,48]. While the Swedish mutation (*K670N/M671L*) used in this study is known to increase total Aβ production by enhancing β-secretase cleavage, we acknowledge that other APP mutations [49], such as the London (V717I) and Florida (I716V) mutations, primarily affect the Aβ42/Aβ40 ratio by altering γ-secretase processing [50]. Although the Swedish mutation provides a robust model for studying amyloid burden and BACE1-related mechanisms, future studies incorporating mutations that specifically alter Aβ42 ratios would provide a more comprehensive understanding of APP processing in AD pathogenesis. Collectively, these data validate the APP–FPN–ferroptosis axis as a critical driver of early AD brain lesions and cognitive impairment, and establish a promising molecular target for AD prevention and treatment [44].

Ferroptosis, a regulated form of cell death driven by iron-dependent lipid peroxidation [51], has been increasingly implicated in neurodegenerative diseases, yet its temporal dynamics and mechanistic link to AD onset remain poorly defined. Our findings clarify that ferroptosis is an early and reversible pathological event in AD, which accounts for the partial restoration of cognitive function upon early intervention [52,53]. The impaired synaptic plasticity and cognitive deficits in *APP/PS1* mice can be attributed to ferroptosis-induced neuronal injury and synaptic degeneration [44,54], consistent with previous studies linking ferroptosis to dendritic atrophy and disrupted neurotransmission [55,56]. Notably, the beneficial effects of ferroptosis inhibition were more pronounced in recognition and in spatial location memory than in spatial learning tasks, indicative of region-specific vulnerability of distinct brain circuits to iron-dependent cell death [52,57]. Additionally, our study revealed that mutant APP reduces membrane FPN abundance, uncovering a regulatory role of APP in maintaining cerebral iron homeostasis. Specifically, the APP *K670N/M671L* mutation causes the decrease of FPN on cell membrane and subsequent aberrant brain iron deposition. While the precise molecular cascade through which APP modulates FPN membrane trafficking warrants further investigation, our data establish a functional link between APP mutation, FPN localization, and ferroptosis in the early stages of AD.

Current AD research and drug development have long been dominated by the Aβ and tau hypotheses, yet nearly all clinical trials targeting these two proteins have failed to meet primary endpoints, indicating the existence of uncharacterized early pathogenic mechanisms [58]. Our study identified an Aβ-independent early pathological cascade, in which membrane FPN instability and ferroptosis activation occur in 2-month-old *APP/PS1* mice without significant Aβ accumulation. We further hypothesize potential crosstalk between this novel axis and classical AD pathways, whereby early ferroptosis-mediated neuronal damage may accelerate Aβ aggregation and tau hyperphosphorylation, forming a pathogenic vicious cycle. This perspective positions iron metabolism dysfunction as an upstream initiator and expands the current framework of AD pathogenesis. Clinically, our findings provide a transformative strategy for early AD intervention, including ferroptosis inhibition, iron chelation, and restoration of APP–FPN binding via peptides. These approaches show high translational potential, supported by the feasibility of non-invasive intranasal Liproxstatin-1 and oral M30 delivery, while also laying the foundation for AD precision medicine via stratified, personalized anti-ferroptosis therapies for patients with brain iron dyshomeostasis.

In our research, the validation of the ferroptosis cascade through three pharmacologically distinct interventions were used: Liproxstatin-1 (Lip-1), M30, and the FTP peptide. Although these agents target different nodes within the ferroptosis pathway, they converge to mitigate AD-like pathology, underscoring the centrality of this pathway in disease initiation. First, these agents represent a “vertical” blockade of the ferroptosis cascade. Lip-1 acts as a radical-trapping antioxidant, scavenging lipid peroxyl radicals at the downstream execution phase of ferroptosis [59]. M30, functioning as an iron chelator, acts at the intermediate level by reducing the intracellular labile iron pool required for the Fenton reaction [60]. By contrast, FTP acts at the most upstream level by targeting the root cause—impaired iron export—by restoring membrane FPN levels and preventing iron overload before it occurs. Despite this mechanistic divergence, the phenotypic convergence is striking. All three interventions effectively suppressed hippocampal lipid peroxidation (reducing MDA and 4-HNE), preserved synaptic integrity, and ameliorated cognitive deficits in *APP/PS1* mice. This “convergence of evidence” demonstrates that, regardless of whether the intervention targets iron influx, iron levels, or oxidative execution, inhibiting the ferroptosis cascade is sufficient to halt the progression of early AD pathology. This robustly validates our primary hypothesis that ferroptosis is not merely a bystander effect but a central driver of neurodegeneration in AD.

A key breakthrough in our findings is the therapeutic mechanism of the APP–FPN binding peptide (FTP). We demonstrate that the *K670N/M671L* mutations impair the interaction between APP and FPN, leading to FPN mislocalization and iron accumulation. The exogenous FTP peptide acts as a “molecular bypass”, directly binding to FPN and restoring its membrane trafficking. This effectively rescues neuronal iron efflux and reverses iron overload. Critically, this restoration of iron homeostasis is the primary driver for the reduction in 4G8 (Aβ) and neuroinflammation. First, iron chelation is a known regulator of APP processing [60]. By restoring FPN-mediated iron export, FTP lowers intracellular labile iron, thereby directly suppressing Aβ production and 4G8 expression. Second, the reduction in neuroinflammation is a consequence of ferroptosis inhibition. Iron overload induces lipid peroxidation (e.g., 4-HNE), releasing DAMPs that activate microglia [61]. FTP-mediated iron chelation halts this peroxidation cascade, reducing oxidative stress and breaking the cycle of inflammation, as evidenced by the downregulation of IL-6 and TNF-α.

Despite the novel insights provided, this study has several limitations. First, our study was conducted exclusively in *APP/PS1* double transgenic mice; future studies are needed to validate the generalizability of the APP–FPN–ferroptosis axis in other AD models (e.g., tau transgenic and sporadic AD animal models). Second, the precise structural domain mediating APP–FPN interactions and the detailed molecular mechanism underlying sAPPα-regulated FPN membrane trafficking remain to be fully elucidated. Third, long-term efficacy assessments, including the effects on chronic neuroinflammation and cerebral atrophy, are necessary to evaluate clinical durability. Additionally, the regional heterogeneity of iron deposition and ferroptosis requires further exploration.

Our study identifies a novel pathogenic mechanism in Alzheimer’s disease (AD) where APP mutations disrupt membrane FPN stability, triggering early ferroptosis. While the Aβ and tau hypotheses have dominated AD research, the failure of clinical trials targeting these proteins underscores the need for new therapeutic strategies.

**Conclusion**: In summary, our data validate the APP–FPN–ferroptosis axis as an early driver of AD pathology. The success of FTP highlights its potential as a therapeutic agent that targets upstream iron dyshomeostasis, offering a promising alternative to traditional Aβ-centric approaches.

## 4. Materials and Methods

### 4.1. Ethics Statements

The mice used in the research were housed and maintained under standard light- and climate-controlled conditions in the Medicine Animal Center of Jianghan University, and the experiments were carried out strictly according to the Medical Ethics Committee of Jianghan University (JHDXLL2023-046).

### 4.2. Animals and Treatment

Wild-type *C57BL/6J* and *APP/PS1* mice (6-week-old) were purchased from Cavens Laboratory Animal Co., Ltd. (Changzhou, China) and maintained until further use. In this study, 2-month-old male *APP/PS1* mice were randomly separated into different groups. Targeted brain delivery of ferroptosis inhibitor (Liproxstatin-1, 2.5 mg/kg/day, Selleck, s7699, Houston, TX, USA) was administered by transnasal administration. Iron chelator (M30, 0.5 mg/kg/day, MCE, HY-131036, Monmouth Junction, NJ, USA) was administered orally. The body weights of the animals were monitored weekly.

Behavioral tests were performed according to the schedule described in the manuscript. A mouse anesthesia mixture (isoflurane + 4% chloral hydrate) was used to anesthetize the mice 24 h after the last behavioral test.

Then the mice were transcardially perfused and the brains were collected for further morphological and biochemical analyses. The tissues were fixed in 4% paraformaldehyde (Biosharp, Beijing, China) for immunofluorescence assays or stored at −80 °C before biochemical analyses.

### 4.3. Plasmid Construction and Cell Treatment

The APP695 gene (NM-201414.3) was inserted into the H312 pEGFP-N1 vector and was named as APP695. Using the forward primer (F: 5′-gtcggaattctgcatccaacttcacttcagagatctc-3′) and reverse primer (R: 5′-gagatctctgaagtgaagttggatgcagaattccgac-3′), we amplified the APP M671L plasmid containing a mutation at position 671, and named it M671L. Using the forward primer (F: 5′-ttctgcatccatattcacttcagagatctcctccg-3′) and reverse primer (R: 5′-cggaggagatctctgaagtgaatatggatgcagaa-3′), we amplified the APP K670N plasmid containing a mutation at position 670, and named it K670N. All plasmid sequences were verified by sequencing analysis. Subsequently, these plasmids were transfected into SY5Y cells to investigate changes in FPN levels in the cell membrane and cytoplasm. Additionally, a membrane and cytoplasmic protein extraction kit (Beyotime Biotech Inc., Shanghai, China) was used to isolate membrane and cytoplasmic proteins separately. The membrane protein marker Na/K^+^-ATPase was used as a loading control for membrane protein samples, while β-actin was used as a loading control for cytoplasmic protein samples.

### 4.4. Stereotaxic Injections of FTP

A peptide fragment of APP that binds FPN (FTP, HRERMSQVMREWEEAERQAKNL) and a scramble peptide (RSQVAERHMEWRKNRMEAEQLE) were obtained from Wuhan Tiande Biotechnology Co., Ltd. (Beijing, China). Peptides were administered via bilateral stereotaxic injection (800 nM, 200 nL, 150 nL/min), as previously described [62]. Briefly, *C57BL/6J* mice and *APP/PS1* mice (8–9 weeks old) were anesthetized with isoflurane and stereotaxically injected with either scramble or FTP peptide. Injections were targeted to the bilateral lateral ventricles (−0.22 mm anterior-posterior, ±1.0 mm medial-lateral, −2.5 mm dorsal-ventral).

### 4.5. Elevated Zero Maze

The elevated zero maze was a circular platform composed of two closed and two open quadrants (XR-XZR209, Shanghai Xinruan Information Technology Co., Ltd., Shanghai, China). The mice were gently placed in the open arm and were allowed to move freely for 10 min. The time spent in the open arm was automatically recorded and analyzed. Between experiments, the platform was cleaned with 75% ethanol.

### 4.6. Barnes Maze

The Barnes maze consisted of a circular open surface (dimensions: diameter = 910mm, height = 1000 mm; Shanghai Xinruan Information Technology Co., Ltd., Shanghai, China). There were twenty circular holes (5 cm in diameter) equally spaced around the perimeter, below which a wooden plastic escape box (11 cm × 6 cm × 5 cm) was placed. Bright light within the room was used to motivate the mice to find the escape box during each session. Moreover, spatial cues consisting of objects in the room were additionally contained to strengthen the space perception of the mice. Between experiments, the platform was cleaned with 75% ethanol to minimize olfactory cues.

The mice were allowed to explore the platform for 60 s during the adaptation phase (Day 0), among which those mice that did not find the escape box were guided to the escape box and remained there for 90 s. During the acquisition phase (Days 1–5), the escape box was kept at the same location and the mice were placed in a start tube located at the center of platform. The tracking system was turned on once the start tube was removed until the mice entered the escape box within 180 s. Those mice that did not enter the escape box within 180 s were guided to the escape box. All the mice remained in the escape box for 60 s after each trial. Primary latency, errors, and primary distance were recorded. On Day 6, the escape box below the escape hole was displaced and each mouse performed one 90-s trial. Primary latency, errors, duration, and entries were measured and recorded.

### 4.7. Open Field Test

For the open field test, mice were placed into an open field box (dimensions: length = 500 mm, width = 500 mm and height = 500 mm; Shanghai Xinruan Information Technology Co., Ltd., Shanghai, China) and were allowed to move for 5 min of free movement. After 4 h, the mice were placed into the same box and monitored by an automated video tracking system for 5 min. The box was cleaned with 75% ethanol to reduce the smell of the other mice between the two experiments. The time spent in the center and the mean speed were analyzed.

### 4.8. Novel Object Recognition Test

After the open field test, the mice were re-introduced into the same open field box, which additionally contained two identical objects (A and B), and were allowed to explore the objects for 10 min. Mice were placed back in their home cages after exploration. One of the two familiar objects (B) in the box was replaced with another novel object (B’) 24 h later. Then the mice were returned to the same box. Mouse behavior was monitored for 10 min. The box was cleaned with 75% ethanol after each trial. The time spent in exploring both the familiar (B) and novel objects (B’) were automatically recorded and analyzed using the Xinruan animal behavior analysis program. The discrimination index was calculated as follows: difference value of time exploring novel (B’) and familiar (B) objects divided by total time [(B’ − B)/(B’ + B)].

### 4.9. Object Location Test

After the novel object recognition test, the objects in the open field box were replaced with another set of two identical objects (C and D), and the position of one object (D) was highlighted by an obvious mark. Then the mice were re-introduced into the box and exposed to the two identical objects (C and D) for 5 min during the acquisition phase. The testing occurred 6 h later in the same box. During the test session, one of the objects (D) was moved to a novel location, and the mice were allowed to explore the objects for 5 min. The box was cleaned with 75% ethanol after each trial. The time mice spent exploring each object was automatically recorded and analyzed. Discrimination index = [(new object location exploration time–old object location exploration time)/total exploration time].

### 4.10. Morris Water Maze

The Morris water maze test is a well-established behavioral paradigm for assessing spatial learning and memory in rodents. The experimental apparatus consisted of a circular pool (160 cm in diameter, 55 cm in height) filled with water maintained at 25 ± 1 °C. The pool was conceptually divided into four equal quadrants (I–IV). A transparent escape platform (12 cm in diameter, 21 cm in height) was submerged 2 cm below the water surface and positioned at the midpoint of quadrant I. To minimize external visual disturbances, the entire pool was surrounded by opaque curtains.

Habituation (Day 0). Twenty-four hours prior to training, the animals were transported to the testing room and allowed to acclimate for at least 30 min. A single pretraining session was conducted, during which each mouse was placed on the visible platform for 20 s and then allowed a 60-s free swim to habituate to the water and experimenter.

Spatial Acquisition Phase (Days 1–6). This phase was designed to evaluate spatial learning and memory acquisition. The experiment was conducted over six consecutive days. Each mouse underwent two training trials per day. For each trial, the mouse was gently placed into the water facing the pool wall, with the starting quadrant varied sequentially in a clockwise direction, always beginning from the quadrant opposite the target (quadrant I). On Day 1, to familiarize the mice with the maze environment, any mouse that failed to locate the platform within 60 s was manually guided to it and allowed to remain there for approximately 10 s. From Day 2 onward, the escape latency—defined as the time taken to locate the hidden platform—was recorded for each trial. If a mouse failed to find the platform within 60 s, its escape latency was recorded as 60 s. The mean escape latency for each group was calculated daily over the 6-day training period and compared between the experimental and control groups to assess spatial learning ability.

Probe Trial (Day 7). This phase was conducted to evaluate spatial memory retention following learning. On Day 7, the platform was removed from the pool. Each mouse was released into the water from the quadrant opposite the original platform location (quadrant I). The number of platform crossings and the time spent in the target quadrant were recorded over a 60-s period. The mean values for each parameter were calculated for both the experimental and control groups and subjected to statistical analysis to compare spatial memory performance.

Data Acquisition and Analysis. All behavioral data, including escape latency, swimming paths, and platform crossings, were automatically recorded and analyzed using a computerized video tracking system. The data collected over the 7-day experimental period were statistically compared among the groups to evaluate differences in spatial learning and memory.

### 4.11. Western Blot

The hippocampus and cortex were rinsed with 1× PBS (phosphate-buffered saline, pH 7.4), minced with scissors, and homogenized on ice. Extraction of whole protein from tissues or cells was performed using RIPA buffer (50 mM Tris, pH 7.4, 150 mM NaCl, 1% TritonX-100, 1% sodium deoxycholate, 0.1% SDS), while extraction of cytoplasmic and membrane protein was performed using the Membrane/Cytosol Protein Extraction Kit (Beyotime Biotech Inc., Shanghai, China). In all buffers, a protease inhibitor cocktail (Beyotime Biotech Inc., Shanghai, China) was used.

The protein concentration in lysates was determined by the BCA Protein Assay Kit (Beyotime Biotech Inc., Shanghai, China). After adding a 5× SDS sample loading buffer, lysates were boiled for 5 min, followed by SDS-PAGE electrophoresis. Then proteins were transferred to nitrocellulose (NC) membranes (Merck Millipore, Darmstadt, Germany) using Mini Gel Tank and Mini Blot Module (Life Technologies, Waltham, MA, USA) and probed using primary antibodies. Blots were visualized using an enhanced chemiluminescence (ECL) system (Bio-Rad, Hercules, CA, USA) after incubation with horseradish peroxidase (HRP)-conjugated secondary antibody (1:3000, Beyotime Biotech Inc., Shanghai, China), and quantified using Image J 1.54r (National Institutes of Health, USA).

### 4.12. Immunohistochemistry

The brain sections were washed in 1× PBS for 3 times, blocked in 5% bovine serum albumin containing 0.1% Triton X-100 (Solarbio, Beijing, China) at room temperature for 1 h, and incubated with primary antibodies at 4 °C overnight. Then the brain sections were washed in 1× PBS for 3 times and incubated with secondary antibody diluted in PBST at room temperature for 1 h. The fluorescence images were obtained using a TCS SP8 STED confocal laser scanning microscope (Leica Camera AG, Wetzlar, Germany) in single-slice mode.

### 4.13. Antibodies

Primary antibodies are described below: mouse anti-β-actin antibody (Boster Bio, Wuhan, China), rabbit anti-synaptophysin antibody (ABclonal, Wuhan, China), rabbit anti-PSD95 antibody (ABclonal, Wuhan, China), rabbit anti-4G8 antibody (Covance, Princeton, NJ, USA), FPN antibody (ABclonal, Wuhan, China), HMGB1 antibody (ABclonal, Wuhan, China), 4-HNE antibody (ABclonal, Wuhan, China), TfR antibody (Abcam, Cambridge, UK), GPX4 antibody (Abcam, Cambridge, UK), Thioflavin S (Shanghai Yuanye Bio-Technology Co., Ltd., Shanghai, China), Na/K^+^-ATPase (Abcam, Cambridge, UK).

### 4.14. Perls’ Prussian Blue Staining

Perls’ Prussian Blue staining was performed as described and with DAB enhancement for detecting iron deposition. Briefly, the brain sections were washed in 1× TBST for 3 times and hydrated with distilled water at room temperature for 5 min. Then the sections were immersed in 3% H_2_O_2_ at room temperature for 10 min. After that, the sections were incubated with a Perl’s mixed solution (4% potassium ferrocyanide/4% hydrochloric acid) at room temperature for 30 min and washed in distilled water for 5 min. Subsequently, 0.025% 3,3-diaminobenzidine (DAB) was used to enhance the iron deposition for analysis. The iron area was measured and quantified using Image-Pro Plus software 7.0.1 (Rockville, MD, USA).

### 4.15. Aβ 1–40 Assay

Fresh cortices were rinsed with 1× PBS (phosphate-buffered saline, pH 7.4), minced with scissors, and homogenized in RIPA buffer containing a protease inhibitor cocktail on ice. After centrifugation, the protein in the supernatant was determined by the BCA Protein Assay Kit (Beyotime Biotech Inc., Shanghai, China). Then, human Aβ 1–40 ELISA kits (Elabscience, Wuhan, China) were used to quantify the absolute Aβ level according to the manufacturer’s instructions. The relative Aβ 1–40 level was calculated by the ratio of OD450 and protein concentration.

### 4.16. Golgi–Cox Staining

After anesthetization, the mice were perfused with 0.9% saline transcardially. The brains were dissected and immersed in the Golgi–Cox solution (5% potassium dichromate, 5% mercuric chloride, and 5% potassium chromate) for 14 days in the dark. After that, the brains were transferred to a 30% sucrose solution at 4 °C until completely immersed and sliced into 70 μm sections using a vibratome (Leica VT1000S, Wetzlar, Germany). Then the slices were transferred onto slides to initiate the staining process. The slices were immersed in ddH_2_O for 1 min followed by incubation in ammonium hydroxide for 30 min. Then, a KODAK Fix solution was applied for another 30 min after the slices were immersed in ddH_2_O for 1 min. The entire process was conducted in the dark. Next, the slices were dehydrated using an increasing grade of ethanol (50%, 75%, 95%, and 100%), then in a CAX solution (xylene/100% ethanol/trichloromethane = 1:1:1) for 15 min, and finally mounted in neutral resin size. A stack of images were acquired from randomly chosen slices on a microscope (Olympus Global, Tokyo, Japan). At least 12 representative images from three to four mice per group were selected for Sholl analysis and dendritic spine analysis.

### 4.17. Measurement of Mitochondrial Membrane Potential (ΔΨm) by JC-1 Staining and Flow Cytometry

JC-1 is a lipophilic cationic dye (Beyotime Biotech Inc., Shanghai, China) that aggregates in healthy mitochondria with high membrane potential (ΔΨm), emitting red fluorescence (590 nm), while it remains as green fluorescent monomers (529 nm) in depolarized mitochondria. The ratio of red to green fluorescence reflects ΔΨm.

After experimental treatment, cells are harvested using a non-enzymatic solution and collected together with floating cells. Cells are centrifuged (300× *g*, 5 min, 4 °C), washed once with cold PBS, and resuspended in warm culture medium at ~1 × 10^6^ cells/mL. An equal volume of JC-1 working solution (diluted in pre-warmed medium to a final concentration of 2 µM) is added, and cells are incubated at 37 °C for 15–30 min in the dark. After incubation, cells are centrifuged, washed twice with cold PBS, and resuspended in 300–500 µL PBS.

Untreated healthy cells serve as a baseline control (high ΔΨm). A positive control is prepared by treating cells with 10 µM CCCP for 30 min to fully depolarize mitochondria, resulting in a green-dominant signal. Unstained cells are used for the autofluorescence setting.

Samples are analyzed on a flow cytometer with a 488 nm laser. Green fluorescence (monomers) is detected in the FITC channel (525–535 nm) and red fluorescence (aggregates) in the PE channel (575–585 nm). At least 10,000 events are acquired per sample. The percentage of cells with decreased red fluorescence (PE) is calculated.

### 4.18. Statistics

Data are displayed as mean ± SEM. All statistical analyses were performed using GraphPad Prism 7 (CA, USA), and *p* value < 0.05 was considered statistically significant. For differences between the two groups, an unpaired two-tailed Student’s *t*-test was used; for differences among multiple groups, one-way ANOVA followed by Tukey’s post hoc test was applied.

## Data Availability

All data supporting the findings in this study are available upon request from the corresponding author.

## Supplementary Materials

The following supporting information can be downloaded at: https://www.mdpi.com/article/10.3390/ijms27093892/s1.

## Abbreviations

The following abbreviations are used in this manuscript:

**Full Name**	**Abbreviations**
Alzheimer’s disease	AD
amyloid-β	Aβ
amyloid precursor protein	APP
ferroportin	FPN
presenilin 1	PS1
transferrin receptor	TfR
malondialdehyde	MDA
prostaglandin-endoperoxide synthase 2	ptgs2
4-hydroxynonenal	4-HNE
glutathione peroxidase 4	GPX4
Liproxstatin-1	Lip1
iron chelator M30	M30
APP-FPN binding domain	FTP
interleukin-6	IL-6
tumor necrosis factor-α	TNF-α
postsynaptic density protein 93	PSD93
phosphate-buffered saline	PBS
radioimmunoprecipitation assay buffer	RIPA
bicinchoninic acid	BCA
sodium dodecyl sulfate-polyacrylamide gel electrophoresis	SDS-PAGE
nitrocellulose	NC
enhanced chemiluminescence	ECL
horseradish peroxidase	HRP
3,3-diaminobenzidine	DAB
